# Raman spectroscopy supported by machine learning reveals changes in balance of macromolecules in diabetic rat serum

**DOI:** 10.1007/s00216-025-06156-9

**Published:** 2025-10-13

**Authors:** Adrianna Kryska, Joanna Depciuch, Mikolaj Krysa, Wieslaw Paja, Agnieszka Wosiak, Marcin Nicoś, Barbara Budzynska, Anna Sroka-Bartnicka

**Affiliations:** 1 Independent Unit of Spectroscopy and Chemical Imaging, Medical University of Lublin, Chodzki 4a, 20-093 Lublin, Poland; 2 Institute of Nuclear Physics, Polish Academy of Sciences, Walerego Eljasza-Radzikowskiego 152, 31-342 Kraków, Poland; 3 Department of Biochemistry and Molecular Biology, Medical University of Lublin, Chodzki 1, 20-093 Lublin, Poland; 4 Department of Artificial Intelligence, Institute of Computer Science, University of Rzeszow, Pigonia 1, 35-310 Rzeszów, Poland; 5 Institute of Information Technology, Lodz University of Technology, Politechniki 8, 93-590 Łódź, Poland; 6 Department of Pneumonology, Oncology and Allergology, Medical University of Lublin, Jaczewskiego 8, 20-090 Lublin, Poland; 7 Independent Laboratory of Behavioral Studies, Medical University of Lublin, Chodzki 4a, 20-093 Lublin, Poland

**Keywords:** Type 2 diabetes, Metabolites, Vibrational spectroscopy, Principal component analysis, Pearson correlation

## Abstract

In type 2 diabetes mellitus (T2DM), changes in glucose and lipid levels in serum are the first signals of disease progression. In this article, Raman spectroscopy supported with machine learning (ML) was used both to determine the level of imbalance between major metabolites caused by the disease and to investigate which metabolites were impacted the most. The principal component analysis separated the T2DM and control rats by PC1 and PC3. The bands that enabled that separation were assigned to all four main metabolite groups—amino acids (745–763 cm^−1^, 985–1027 cm^−1^), amides (1199–1258 cm^−1^, 1574–1600 cm^−1^, 1653–1712 cm^−1^), polysaccharides (1104–1192 cm^−1^), and lipids (1442–1472 cm^−1^, 2831–3041 cm^−1^). Moreover, diabetes induction caused major correlation changes between both proteins (amide) and the general amino acid band, with the organic matter band (CH band), which mainly arises due to lipid presence. Moreover, four different machine algorithms were employed to detect changes between the groups. AdaBoost showed the best performance in classifying the serum samples from diabetic and control rats (*F*_1_ = 0.84), which was further refined when only the relevant bands were used for learning and classification (F_*1*_ = 0.94). The bands responsible for the separation suggest that T2DM induces alterations in the protein profile, including changes in the levels of aromatic amino acids, as well as in both the quantity and composition of lipids, which is further confirmed by the ML classification.

**Supplementary Information:**

The online version contains supplementary material available at 10.1007/s00216-025-06156-9.

## Introduction

In the past decades, type 2 diabetes mellitus (T2DM) has been posing a rising threat to public health. Due to environmental and social factors, the prevalence of T2DM has been increasing drastically, with estimated cases of 463 million in 2019. The number is predicted to reach up to 700 million within the next decade [1, 2]. T2DM has been described as a group of metabolic disorders, where the absence of treatment leads to abnormally high glucose levels in blood (hyperglycemia) [3]. The development of T2DM is related to combined dysfunction of β cells of pancreatic islets and decreased sensitivity of insulin-dependent cells [3, 4]. When the previously mentioned cells become insulin-resistant, glucose transport to the tissues becomes impaired and causes hyperglycemia [5]. Hyperglycemia and insulin resistance do not present visible symptoms in the early stage of the disease. For this reason, the diagnosis is usually made at the onset of complications (which include retinopathy, nephropathy, coronary heart disease, or lipid metabolism disorders [2]). Therefore, developing a non-invasive and rapid technique to detect diabetes-related complications at an early stage is crucial. Diabetes diagnosis is established through two main tests: the level of fasting plasma glucose and the level of plasma glucose after 2 h from 75 g of oral glucose intake [3]. However, it is noteworthy that a blood test has limitations as it only partially shows how the disease develops and cannot reveal changes occurring in organs. In recent years, the analysis of body fluids, including blood serum, with Raman spectroscopy has been studied [6]. The sensitivity of the technique and the ability to detect changes in chemical composition make it a useful tool for the diagnosis, therapeutic monitoring, and further analysis of the disease [7, 8]. Raman spectroscopy analyzes molecular vibrations unique to a compound’s structure through inelastic light scattering. When a monochromatic laser illuminates a sample, photon interactions cause the light to scatter and the energy of some photons shifts. These shifts form a Raman spectrum, where band positions and intensities reveal molecular composition. The vibrational energy depends on atomic masses, bond strength, and environmental interactions. This makes Raman spectroscopy a powerful tool for detecting molecular and chemical changes, including those caused by diseases or external substances [9, 10].

In this work, Raman spectroscopy was used to determine changes in the chemical composition in the serum caused by T2DM. To deepen the insights, the correlation between lipids and other groups of compounds in the diabetic model was also investigated. Furthermore, to demonstrate the differentiation of samples collected from the two groups, both unsupervised (principal component analysis, PCA) and supervised machine learning techniques (decision tree, k-nearest neighbors, random forest, and AdaBoost) were applied. These methods were also used to extract spectral features to provide deeper insight into the chemical changes associated with T2DM.

## Materials and methods

### T2DM model

In this study, experiments were conducted on 6-week-old drug-naïve male Wistar rats. Their weight at the beginning of the experiment was 180–220 g. The animals were obtained from the Centre of Experimental Medicine, Medical University of Lublin. Thirty rats were divided into two experimental groups: 20 for the diabetic model and 10 for the control group. The animals were placed in sex-matched pairs with a 12-h light/dark cycle (with the light cycle starting at 8 a.m.), a room temperature of 21 ± 1 °C, and a relative humidity of 50 ± 5%. Throughout the experiment, the control group was fed standard rodent feed and the diabetic group was fed a high-fat diet (HFD, EF D12451, 45% kJ% Fat; Ssniff.). The experiments were conducted in accordance with the “Animal Research: Reporting In Vivo Experiments’ (ARRIVE)” guidelines. All experiments were carried out following the National Institute of Health Guidelines for the Care and Use of Laboratory Animals and the European Community Council Directive for the Care and Use of Laboratory Animals of 22 September 2010 (2010/63/EU) and were approved by the Local Ethics Committee in Lublin, Poland (Permission No: 14/2022).

The model for T2DM was established by using HFD for inducing insulin resistance and a single streptozotocin (STZ—35 mg/kg) injection for β cells of pancreatic islets’ dysfunction [11].

STZ was dissolved in a citrate buffer, adjusting the pH to 4.5, the most suitable pH for the injection. The solution was prepared immediately before injection and used within 5 min from preparation. STZ has maximum stability at pH 4; however, it degrades in the citrate buffer after approximately 15–20 min. Due to the STZ sensitivity to light, the solution tubes were covered with aluminum foil for protection. Animals were fasted for 4 h before STZ injection [12].

The experiment lasted 50 days. Glucose levels were measured on the 1 st, 21 st, 36th, and 50th days. On the 14th day, a single dose of 35 mg/kg STZ was injected. If the rats exhibited fasting glucose levels exceeding 150 mg/dL on the 50th day, they were classified as diabetic, and only those were selected for further experiments. On the last day of the experiment, the rats were decapitated and blood samples were collected at a volume of approximately 150–200 µL. The blood samples were centrifuged at 4 °C for 10 min at 3000 rpm. The serum was collected in 0.2-mL Eppendorf tubes and stored at −80 °C for further analysis.

### Raman measurements

In this study, 20 samples of blood serum were measured, 10 from the diabetic model and 10 as control. From each sample, 4-μm droplets of serum were placed on CaF_2_ Raman grade slides and left for approximately 15 min to dry. For the measurements, the WITec alpha300 R Raman imaging microscope (WITec GmbH, Ulm, Germany) was used. The spectra were collected with a laser excitation of 532 nm, 7 mW laser power, and a 100× objective. Each spectrum was accumulated 12 times with an integration time of 1 s. Five spectra were collected from each serum sample (from a different place of the dried serum). After collection, the spectra were cropped to the 400–3500 cm^−1^ range, cosmic rays were removed, and baseline correction was applied using Shape mask with a value of 170 applied with WITec Project SIX software. Lastly, area normalization was performed for each obtained spectrum, followed by the removal of Raman shifts in the 1800–2800 cm^−1^ range using Quasar software (ver. 1.10.2).

### Analysis of Raman spectra

All of the below-mentioned analyses were performed on the 10 control and 10 diabetic samples, each of which was collected 5 times; therefore, on 100 spectra.

Principal component analysis (PCA) was performed using five principal components on scaled data. The components with the greatest group separation, as indicated by the lowest *p*-values, were selected for further analysis. Loading plots were generated, highlighting values exceeding ± 0.4 to denote the primary contributors to the group separation. The analyses were conducted in Python 3.12.7.

Pearson correlation analysis was utilized to examine the relationships between lipid band areas and other spectral bands, with *p*-values and correlation coefficients used to determine the strength and significance of the correlations. This analysis was carried out using Python 3.12.7.

To classify diabetic metabolic information from serum Raman spectra, four ML algorithms were evaluated: (a) decision tree (DT) using the Gini criterion; (b) k-nearest neighbors (*k* = 7); (c) random forest (RF) with the Gini criterion and 100 estimators; (d) AdaBoost (AB) with a decision tree classifier (Gini criterion, 100 estimators, max depth = 1). Each algorithm was executed 10 times with optimized parameters, and the results were averaged to reduce the influence of random variation. The most influential bands identified by RF and AB were employed to refine the classification model. A leave-two-samples-out cross-validation method ensured unbiased training and testing. Moreover, the decision tree was extracted to analyze which bands impacted it the most. For interpretation, the F1 parameter was used (harmonic mean of the precision and recall of a classification model). All of the above-mentioned analyses were implemented using Python 3.12.7 with the scikit-learn library.

## Results

**Fig. 1:**
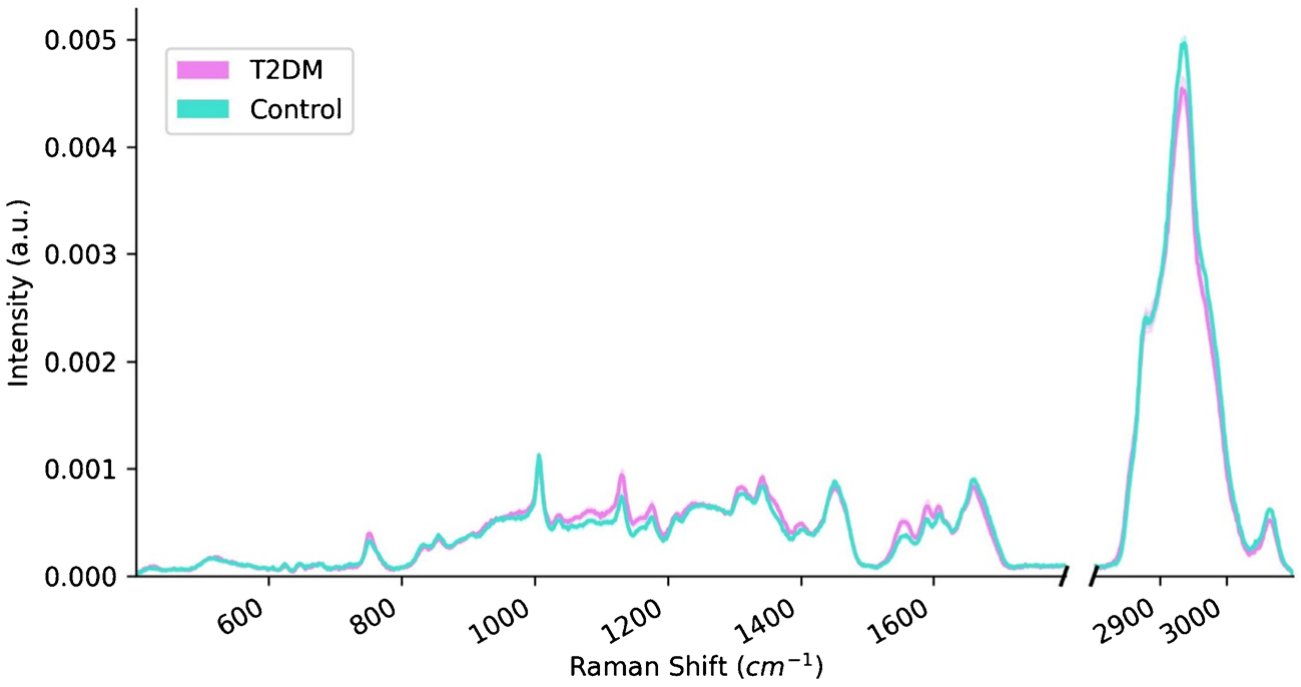
Mean spectra of serum collected from diabetic (T2DM) and control groups

In this study, Raman spectra were analyzed to investigate the changes in the level of the metabolites in the serum caused by T2DM. Serum spectra from both control and T2DM rats exhibited vibrations corresponding to the same functional groups (Fig. 1a). In Fig. 1a, bands originating from C-O-H and CO-C polysaccharides functional groups were visible in the ranges 1104–1152 cm^−1^ and 1148–1192 cm^−1^, respectively [13, 14]. Moreover, the amides vibrations were also observed in the Raman shift ranges 1199–1293 cm^−1^ (amide III), 1574–1600 cm^−1^ (amide II), and 1629–1712 cm^−1^ (amide I) [15]. The disulfide band arising due to the protein tertiary structure was observed in the range 477—604 cm^−1^ [16]. Lipid vibrations were visible in the 1330–1388 cm^−1^ (CH_3_ bending), 1422–1494 cm^−1^ (CH_2_ bending), and 2818–3041 cm^−1^ (CH stretching) ranges [15, 17–19]. Furthermore, vibrations of amino acids were present in the ranges 727–791 cm^−1^ (skeletal and ring vibrations of amino acids), 812–873 cm^−1^ (tyrosine out-of-plane ring breathing), and 985–1027 cm^−1^ (phenylalanine symmetric ring breathing) [20, 21].

**Fig. 2:**
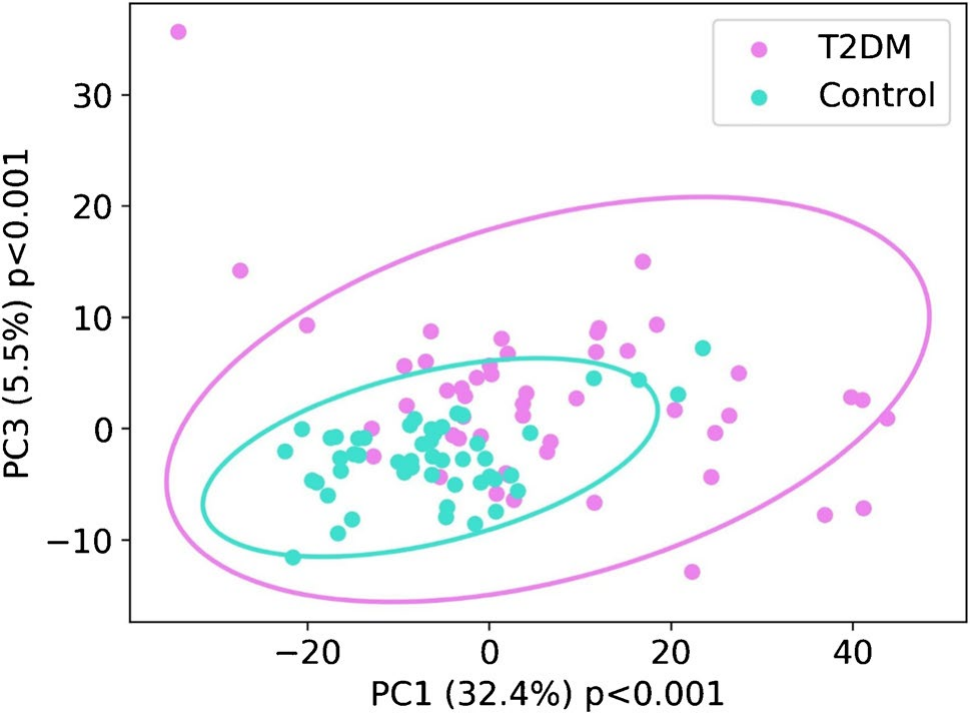
Principal component analysis plot from the spectra of T2DM and control serum samples. Component 1 is plotted against component 3. “*p*” near both axes represents *p*-values of the statistical analysis of the scores of T2DM and control groups on each component. The graph shows sufficient separation between the two groups. PCA was performed, and 95% confidence intervals were calculated around the PCA clusters

To illustrate the potential application of Raman spectroscopy in distinguishing serum from control and T2DM rats, principal component analysis (PCA) was conducted (Fig. 2). After selecting PC1 and PC3 components as showing the greatest separation, PCA revealed quite sufficient differentiation between the two groups.

**Fig. 3:**
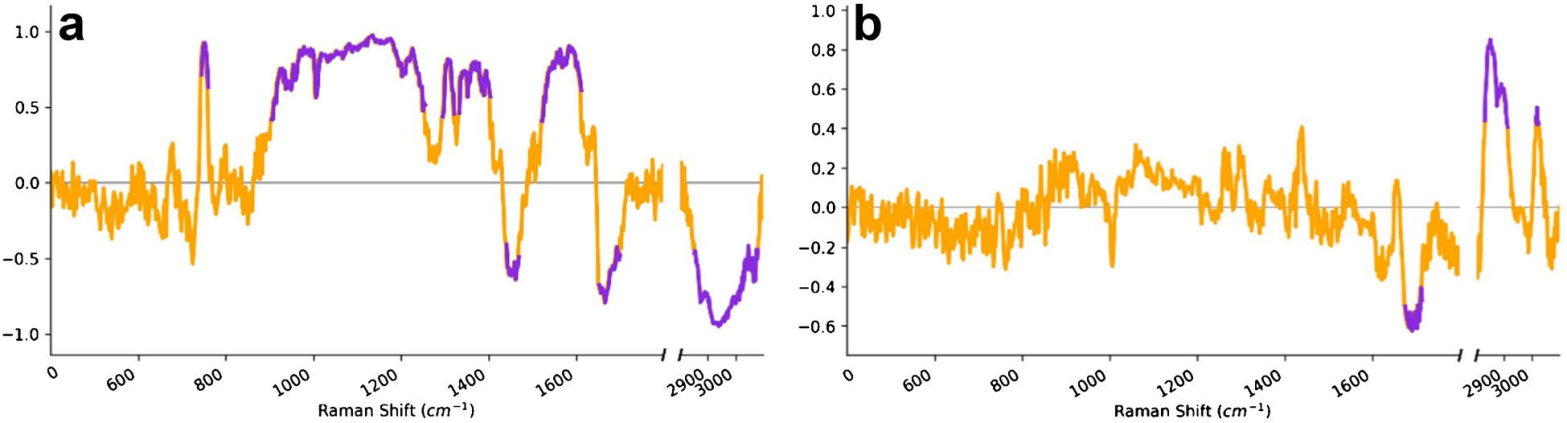
Loading plots obtained from PCA, presenting Raman shifts, which significantly contributed to the differentiation between samples. **a** PC1 loading plot; **b** PC3 loading plot

PCA loading plots were utilized to identify the Raman shifts that contributed most significantly to the observed separation (Fig. 3). The loading plot of PC1 exhibited that the bands in the ranges: 745–763 cm^−1^, 905–1258 cm^−1^, 1296–1324 cm^−1^, 1442–1472 cm^−1^, 1523–1614 cm^−1^, 1653–1703 cm^−1^, and 2852–3081 cm^−1^ separated the T2DM from control samples by PC1 score the most significantly. On the other hand, the loading plot of PC3 showed that the bands in the ranges of 1676–1716 cm^−1^, 2831–2914 cm^−1^, and 3015–3026 cm^−1^ accounted for the separation by the PC3 scores. These findings suggest that the identified metabolites changed the most significantly in response to T2DM.

In order to evaluate the correlation between the bands in both of the groups, Pearson correlation was performed and results have been presented in Table 1. The analysis revealed distinct differences in spectral band correlations between the control and T2DM groups. A positive correlation was observed between the lipid-associated band (1330–1388 cm⁻^1^) and all other analyzed bands except amide I (1629–1712 cm^−1^). Negative correlations were found between the 2818–3041 cm⁻^1^ lipid band and all other bands except amide I (1629–1712 cm^−1^) and protein tertiary structure (477–608 cm⁻^1^) bands, with stronger negative correlations in the T2DM group. In the control group, the strongest correlation was displayed between the 1330–1388 cm^−1^ band (lipids) and the 1199–1293 cm^−1^ band (amide III); however, it weakened in the T2DM group. Key differences between the groups also included stronger correlations between 1330–1388 cm⁻^1^ (lipids) and 1599–1627 cm⁻^1^ (amide I) and 1517–1573 cm⁻^1^ (amide II) in the T2DM group. A significant decrease in correlation was noted between 1422–1494 cm⁻^1^ (lipids) and 1629–1712 cm⁻^1^ (amide I) in the diabetic group (from 0.68 to 0.38). The correlation between 2818–3041 cm⁻^1^ (lipids) and 477–608 cm⁻^1^ (protein tertiary structure) also weakened significantly in the T2DM group (from 0.68 to 0.25). Overall, the T2DM group exhibited stronger correlations, with additional significant correlations absent in the control group, suggesting alterations in lipid-protein interactions.

**Table 1:** Pearson correlation between bands corresponding to lipids and polysaccharides, amides, and amino acids in serum collected from control and T2DM rats. Strong correlations (> 0,6) are marked with ** on the right side of the value, weak correlations (< 0,4) are marked with ^#^ on the right side of the value. Statistically significant correlations are marked with * on the left side of the value

		T2DM			Control		
		Lipids					
	cm^−1^	1330–1388	1422–1494	2818–3041	1330–1388	1422–1494	2818–3041
Amides	1629–1712	* −0.441	* 0.384^#^	* 0.335^#^	* −0.445	* 0.676**	* 0.550
	1599–1627	* 0.917**	* 0.005^#^	* −0.645**	* 0.687**	* 0.291^#^	−0.004^#^
	1574–1600	* 0.853**	* −0.304^#^	* −0.805**	* 0.850**	−0.198^#^	* −0.467
	1517–1573	* 0.929**	* −0.142^#^	* −0.699**	* 0.827**	−0.175^#^	* −0.510
	1199–1293	* 0.807**	* −0.186^#^	* −0.664**	* 0.911**	0.069^#^	−0.162^#^
Polysaccharides	1148–1192	* 0.787**	* −0.483	* −0.846**	* 0.790**	* −0.404	* −0.626**
	1104–1152	* 0.742**	* −0.532	* −0.857**	* 0.714**	* −0.513	* −0.706**
Amino acids	985–1027	* 0.670**	* −0.541	* −0.821**	* 0.644**	* −0.559	* −0.740**
	727–791	* 0.792**	* −0.276	* −0.774**	* 0.835**	0.155^#^	−0.113^#^
Protein tertiary structure	477–608	0.058^#^	* 0.454	* 0.254^#^	* 0.280^#^	* 0.666**	* 0.680**

In order to examine whether the chemical changes caused by T2DM are significant enough to enable proper classification of the samples, four ML algorithms were used: decision tree (DT); random forest (RF); AdaBoost (AB), and k-nearest neighbor (kNN). Based on obtained results, the AdaBoost algorithm displayed the highest values for *F*_1_ (0.84), accuracy (0.85), sensitivity (0.80), specificity (0.90), and precision (0.89) (the result table is presented in Supplementary Materials (S1)).

**Fig. 4:**
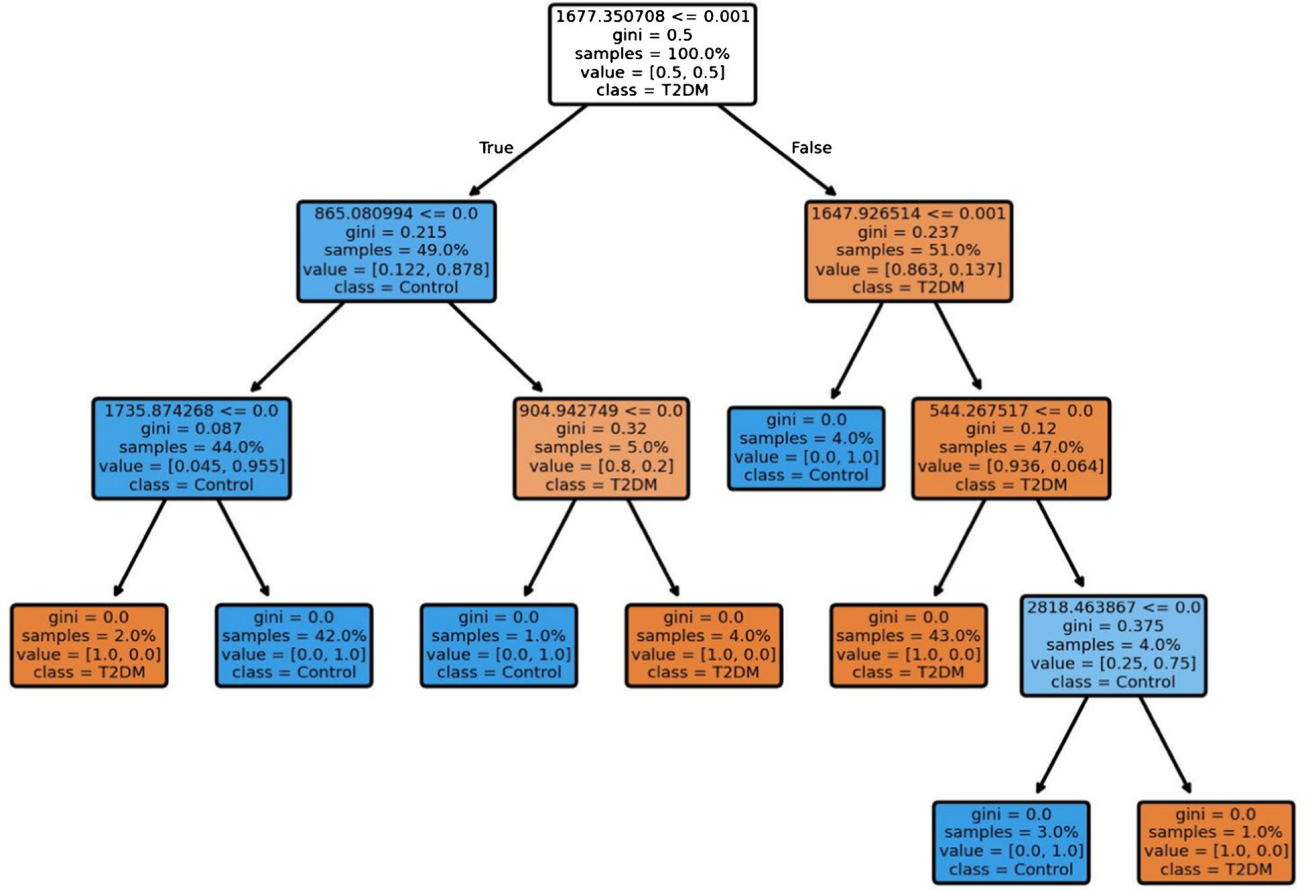
Diagram of the decision tree from the Raman spectra of T2DM and control serum. Each decision node has an information about (from the top): the criterion of the node (Raman shift with the intensity); Gini score—the function to measure the quality of a split; the percentage of the samples from the whole analyzed samples; the ratio of the samples belonging to T2DM and control group respectively; the dominating class of samples. The orange color represents the nodes that are dominated with T2DM samples, while blue represents the nodes that are represented with control samples

Based on obtained results, the decision tree is presented in Fig. 4, which indicated that shifts at 544 cm^−1^, 865 cm^−1^, 1648 cm^−1^, and 1736 cm^−1^ show the highest rate of classification and could be used as a determinant of diabetes; however, with less accuracy than other ML algorithms.

**Fig. 5:**
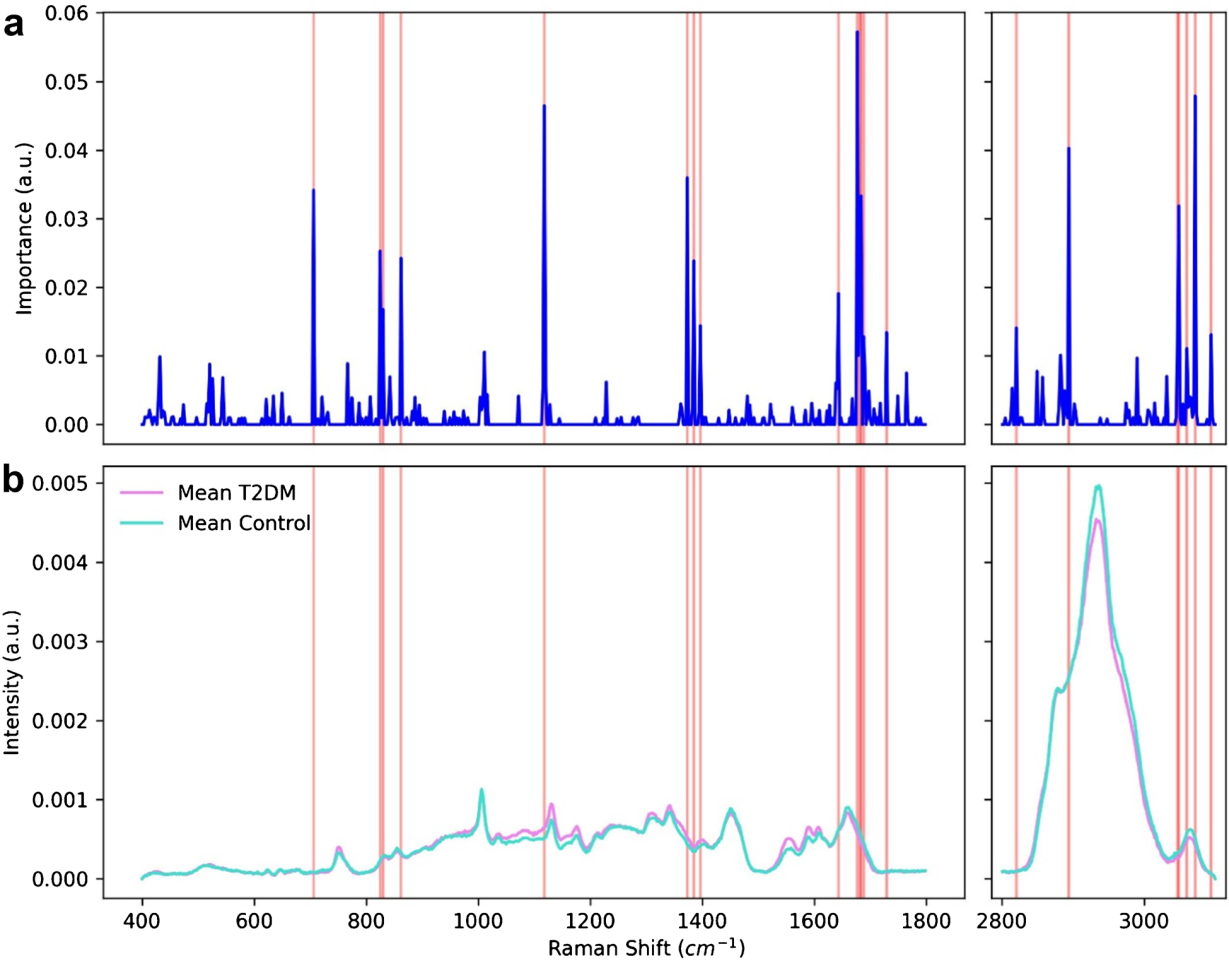
Plot representing the importance of the Raman shifts, to classify the spectra using AdaBoost algorithm (**a**), and the corresponding mean serum spectra of diabetic and healthy rats (**b**). The bands that contributed the most to the classification and that were used to refine the classification are marked in red

Moreover, since AdaBoost was the most successful algorithm in distinguishing between control and diabetic rats, the bands that impacted the classification the most could be extracted (Fig. 5). The most significant changes that influenced the classification were assigned to the amide I band (1640 cm^−1^–1680 cm^−1^ range), S-S stretching of protein tertiary structure (544 cm^−1^), stretching vibrations of CH_2_ and CH_3_ (2800–3100 cm^−1^), amino acids such as tyrosine and phenylalanine (767 cm^−1^, 825–863 cm^−1^, and 1118 cm^−1^), the band at 706 cm^−1^ assigned to deformation vibrations of the cholesterol ring, 1373, 1385, and 1397 cm^−1^, which may be assigned to lipid CH_3_ bending, and the C=O stretching of esters (1729 cm^−1^). All Raman shifts with importance values were included in supplementary S1.

To further verify that T2DM mostly impacts those metabolic changes, another ML analysis was performed on 22 Raman shifts, indicated as relevant by the AdaBoost algorithm.

The analysis revealed that the values of the accuracy, sensitivity, specificity, and precision were over 89% for RF, AB, and kNN, and 78% for DT (Table 2). This significant improvement in the classification parameters of all the algorithms further confirms that those 22 Raman shifts, indicated as relevant by the AdaBoost algorithm, are indeed the most significant changes caused by T2DM in the rat serum.

**Table 2:** Classification results between control and T2DM groups obtained with four different ML after selection of the most important Raman shifts

	*F*_1_	Accuracy	Sensitivity	Specificity	Precision
Decision tree	0.79	0.79	0.79	0.79	0.79
Random forest	0.90	0.90	0.90	0.90	0.90
AdaBoost	0.94	0.94	0.94	0.94	0.94
K-nearest neighbor	0.93	0.93	0.92	0.94	0.94

## Discussion

Extremely high prevalence of diabetes, particularly type 2 diabetes mellitus (T2DM), underscores the critical need for in-depth investigation into the disease’s pathophysiological mechanisms and the identification of novel biomarkers for predicting the risk of complications. Current literature emphasizes the significant role of lipids and alterations in polysaccharide metabolism in the disease’s progression [22, 23]. This study aimed to explore the lipid, polysaccharide, and protein metabolic changes in the context of diabetes. Elevated glucose levels in the blood serum of diabetic patients are well documented and are associated with disruptions in lipid homeostasis, culminating in dyslipidemia [24, 25]. Our findings highlight a significant correlation between amides and lipids, suggesting that both compound groups are crucial in the pathophysiology of diabetes. This correlation is notably stronger in T2DM rats compared to controls, likely due to disruptions in protein and lipid balance. Our results also presented a stronger correlation between lipids and polysaccharides in the T2DM group, indicating the significance of those compounds in diabetes. Chronic hyperglycemia and dysregulation of lipid profiles, including cholesterol, low-density lipoproteins (LDL), high-density lipoproteins (HDL), and triglycerides, are characteristic in diabetes [26]. In other studies, where Raman spectroscopy was used to determine changes in the polysaccharide and lipid fractions in diabetes, Fernandes Borges et al. presented a model based on PCA. They showed that by using Raman spectroscopy it is possible to determine glucose and LDL levels [27]. In diabetes mellitus, an increase in low-density lipoprotein (LDL) and triglyceride levels and a decrease in high-density lipoprotein (HDL) concentrations are observed [28]. Raman spectroscopy has been used in diabetes research to monitor changes in glucose levels, particularly for sensor applications [29]. Additionally, several studies have explored its applicability in the screening of diabetes mellitus [30] and in distinguishing between healthy and diabetic patients [31]. Duc et al. applied principal component analysis (PCA) in conjunction with a support vector machine (SVM) model to evaluate the accuracy of Raman spectroscopy in patients with diabetes, achieving an accuracy rate of 80% [31]. Another study by Guevara et al. analyzed Raman spectra from different body sites (ear lobe, inner arm, thumbnail, and median cubital vein) in individuals with T2DM and a control group, where ML techniques were used for classification. An artificial neural network (ANN) classifier achieved high accuracy values, outperforming fasting capillary blood glucose testing and matching the gold standard fasting venous plasma glucose in sensitivity but with lower specificity. Moreover, correlation analysis linked Raman spectral components to advanced glycation end-products (AGEs) across various sampling sites [32]. In our study, we employed ML algorithms not only for classification but also for detection of the most significant changes, which pointed at amides, amino acids like tryptophan and phenylalanine, and lipids (mainly phospholipids and cholesterol). Additionally, we achieved high levels of classification parameters (> 96% for AdaBoost when using only the most relevant bands), exceeding those achieved in other studies. PCA presented in Fig. 2 and ML models in Table 2 revealed the ability for high differentiation between serum collected from control and T2DM rats. The decision tree also indicated that the band at 1648 cm^−1^, which originates from ν C=O vibrations in amide I, might be a candidate for a Raman marker of the disease (Fig. 5).

These findings suggest that Raman spectroscopy shows not only differentiation between control and T2DM groups but also reveals changes in the polysaccharide-lipid-protein balance in diabetes, which could cause the complications of the studied disease. However, there is still a need for further research to understand the metabolic changes in T2DM.

## Conclusions

In this study, diabetes-induced disruption in the balance of metabolites in the rat serum was detected and further investigated. The in-depth analysis showed a change in correlation strength between the analyzed groups of chemical compositions, which could suggest that changes in the balance between polysaccharides and lipids, as well as between amides and lipids, occurred during diabetes. The results also revealed that PCA enables differentiation of serum collected from control and T2DM groups of rats, based on the changes occurring in amino acids, amides, and lipids. Analysis using ML algorithms showed that the models built from Raman data demonstrate high quality in classification and recognition of animal cases. Most of them allow obtaining a classification accuracy of more than 0.82 with similar values of other parameters such as sensitivity, specificity, and precision. Those parameters were further refined to over 0.9 when only relevant bands were used. AdaBoost was the most effective algorithm in distinguishing between control and T2DM rats, allowing the identification of key spectral bands influencing classification. The most significant changes were observed in proteins, amino acids like phenylalanine and tyrosine (745–763 cm^−1^, 985–1027 cm^−1^, 1199–1258 cm^−1^, 1574–1600 cm^−1^, 1653–1712 cm^−1^), as well as lipid-related bands (1442–1472 cm^−1^, 2831–3041 cm^−1^). It indicates that diabetes results in a strong disruption in the homeostasis of the metabolites in the serum.

## Supplementary Information

Below is the link to the electronic supplementary material.

Supplementary Material 1 (DOCX 17.2 KB)

## Data Availability

The datasets generated during and/or analyzed during the current study are available in the Mendeley Data repository 10.17632/t3w576ykdf.2 [33].
