# Supplementary material for: Raman spectroscopy supported by machine learning reveals changes in balance of macromolecules in diabetic rat serum
Source: Anal Bioanal Chem. 2025 Oct 13;417(29):6655–63. doi: 10.1007/s00216-025-06156-9 (PMC12641036; doi:10.1007/s00216-025-06156-9)
Supplement: Supplementary file 1 — Supplementary Material 1 (DOCX 17.2 KB) [file 216_2025_6156_MOESM1_ESM.docx]

Table S1 Classification results between control and diabetic groups obtained with four different ML algorithms.

|  | F1 | Accuracy | Sensitivity | Specificity | Precision |
| --- | --- | --- | --- | --- | --- |
| Decision Tree | 0.768 | 0.757 | 0.804 | 0.710 | 0.735 |
| Random Forest | 0.813 | 0.822 | 0.774 | 0.870 | 0.856 |
| AdaBoost | 0.842 | 0.850 | 0.800 | 0.900 | 0.889 |
| K-Nearest Neighbor | 0.826 | 0.840 | 0.760 | 0.920 | 0.905 |

Table S2 Raman shifts with importance values obtained with AdaBoost algorithm.

| Wavenumber [cm^-1^] | Importance |
| --- | --- |
| 544 | 0.0115 |
| 706 | 0.0285 |
| 767 | 0.0126 |
| 825 | 0.0259 |
| 830 | 0.0200 |
| 863 | 0.0218 |
| 1118 | 0.0429 |
| 1373 | 0.0329 |
| 1385 | 0.0232 |
| 1397 | 0.0136 |
| 1643 | 0.0182 |
| 1677 | 0.0632 |
| 1680 | 0.0121 |
| 1682 | 0.0271 |
| 1684 | 0.0403 |
| 1689 | 0.0136 |
| 1729 | 0.0142 |
| 2820 | 0.0147 |
| 2894 | 0.0468 |
| 3047 | 0.0142 |
| 3049 | 0.0184 |
| 3071 | 0.0505 |
